# tsRNA-15797-modified BMSC-derived exosomes mediate LFNG to induce angiogenesis in osteonecrosis of the femoral head

**DOI:** 10.55730/1300-0152.2654

**Published:** 2023-05-18

**Authors:** Shanhong FANG, Mengqiang YOU, Jie WEI, Peng CHEN

**Affiliations:** 1 Department of Sports Medicine, National Regional Medical Center, Binhai Campus of the First Affiliated Hospital, Fujian Medical University, Fuzhou, China; 2 Department of Orthopedic Surgery, the First Affiliated Hospital, Fujian Medical University, Fuzhou, China

**Keywords:** Osteonecrosis of the femoral head, tsRNA-15797, exosomes, angiogenesis

## Abstract

**Background:**

Osteonecrosis of the femoral head (ONFH) is an ischemic disease characterized by the impairment of angiogenesis. We have previously elucidated the role of tsRNAs and BMSC exosomes in ONFH, but whether tsRNA-modified BMSC exosomes promote angiogenesis in ONFH remains unclear.

**Methods:**

The expression of angiogenesis-related tsRNA in plasma exosomes from ONFH patients was examined by q-PCR. The function of tsRNA in HUVECs was identified by CCK-8 and angiogenesis assay. Exosomes purified from tsRNA-15797 overexpressed BMSCs were cocultured with HUVECs to examine their role in angiogenesis. The molecule mechanism of tsRNA-15797-modified exosomes was explored by RNA sequencing, dual-luciferase assay, and immunofluorescence.

**Results:**

A tRNA-derived small RNA tsRNA-15797 was down-regulated in plasma exosomes of ONFH patients. We found the effects of BMSCs-derived exosomes on accelerating HUVECs angiogenesis and migration, which were further enhanced after overexpressing tsRNA-15797. Besides, overexpression of tsRNA-15797 would lead to down-regulation of LFNG correlated with angiogenesis. tsRNA-15797 could directly interact with LFNG. We demonstrated that LNFG overexpression weakened the pro angiogenic and migratory effects of tsRNA-15797-modified BMSCs-derived exosomes.

**Conclusion:**

We successfully constructed tsRNA-15797-modified BMSC-derived exosomes and demonstrated that it induced the angiogenesis of HUVECs by targeting the down-regulation of LFNG. Thus, tsRNA-15797-loaded BMSCs-derived exosomes may be a potential target therapy drug for ONFH.

## 1. Introduction

Osteonecrosis of the femoral head (ONFH) is a common refractory disease in clinical practice, in which the arterial blood supply of the femoral head is damaged or interrupted, which leads to the death and subsequent repair of osteocytes and bone marrow components, and then leads to structural changes of the femoral head, the collapse of the femoral head, and joint dysfunction (Wang et al., 2018). ONFH can be caused by a variety of causes, such as long-term use of glucocorticoids, alcoholism, hip trauma, rheumatic diseases, and atherosclerosis (Yuan et al., 2016). Currently, the treatment of ONFH is mainly divided into two types, one is a nonsurgical treatment, such as drug therapy and electrical stimulation, and the other is a surgical treatment, such as bone grafting (Tong et al., 2018; Zhao et al., 2020). However, although there are many treatments for ONFH, there is still no cure. In view of this, it is urgent to explore the pathogenesis of osteonecrosis of the femoral head and develop new treatment techniques.

Bone marrow mesenchymal stem cells (BMSCS) are bone marrow stromal stem cells with multiple potentials to differentiate into bone, cartilage, fat, nerve, and myoblasts (King et al., 2014; Yu et al., 2020). The disordered osteogenesis and adipogenesis of BMSCs caused the defeat of repair of osteocytes during osteonecrosis (Zhu et al., 2020). At present, mesenchymal stem cells are widely used in the treatment of ONFH. For example, BMSCs can inhibit the deterioration of early ONFH (Zhao et al., 2012). In addition, weakening the mineralization and osteogenic suppression of hBMSCs is an important direction for the treatment of ONFH (Yang et al., 2019). In fact, the therapeutic capacity of BMSCs depends mainly on secreted regulatory vectors, such as exosomes. Exosomes are extracellular vesicles that mainly transfer proteins and RNAs (miRNA, etc.) between cells (Gu et al., 2020). Exosomes secreted by BMSCs regulated cell survival and tissue repair (He et al., 2020). Meanwhile, in our previous study, BMSCs-secreted exosomes had a protective effect on steroid-induced osteogenesis of ONFH (Fang et al., 2018; Fang et al., 2020). However, the regulatory mechanisms involved remain unclear.

tRNA-derived small RNAs (tsRNAs) are a new class of noncoding RNA found in many organisms (Falconi et al., 2019). tsRNAs play an important role in the occurrence and development of various diseases (Zong et al., 2021). Most importantly, due to their stable structure, tsRNAs are distributed in peripheral tissues, body fluids and exosomes, and can be regarded as a clinical biomarker or therapeutic target (He et al., 2021). For instance, we have found abnormal expression profiles of tsRNA in plasma of ONFH, in which BMSC-derived exosomes loaded with tsRNA-10227 promote osteogenesis of ONFH (Fang et al., 2020). Therefore, exploring the regulatory function of tsRNA-modified BMSC exosomes on angiogenesis will contribute to the treatment of ONFH.

Our study aims to identify beneficial exosomal tsRNA, construct tsRNA-modified BMSC-derived exosomes, and verify their efficacy in the treatment of ONFH by function and mechanism related experiment in vitro, which is expected to explore new molecular therapy tool for ONFH.

## 2. Materials and methods

### 2.1. Human plasma specimens

The six subjects included were divided into control group (healthy individuals) and experimental group (ONFH patients), with three subjects in each group. After anticoagulant treatment, the blood samples were centrifuged at 1000 rpm for 15 min at 20 °C to collect plasma. Samples were stored at −80 °C for subsequent experiments. None of the ONFH patients obtained any treatment prior to blood collection.

### 2.2. Cell culture

The Procell Life Science & Technology Co. Ltd. (Wuhan, China) provided human BMSCs and human umbilical vein endothelial cells (HUVECs). BMSCs were cultured in RPMI-1640 media (GIBCO) containing 10% fetal bovine serum (FBS), and HUVECs were cultured in ECM medium with 10% FBS. All cells were cultured in an incubator at 37 °C, 5% CO_2_. After 72 h of incubation, the entire medium was replaced, and every 2–3 days thereafter. Cell passage can be performed when the cells are 80% full of the cell culture flask (Corning Inc., USA).

### 2.3. Isolation and identification of exosomes

ExoQuick™ Plasma Pretreatment and Exosome Precipitation Kit (SBI, EXOQ5TMA-1, Japan) was applied to isolate plasma exosomes from ONFH patients and healthy individuals. Briefly, plasma samples were collected and added SBI Thrombin reagent for incubation at room temperature for 5 min, then centrifuged at 10,000 rpm for 5min at 4 °C. Subsequently, 63 μL ExoQuick Exosome Precipitation Solution was added to 250 μL supernatant and allowed to precipitate for 30 min at 4 °C. After centrifugation at 1500 × g for 5 min at 4 °C, the exosomes were resuspended in sterile 1 × PBS for precipitation and stored in a −80 °C refrigerator.

Ultra-high speed centrifugation was applied to extract exosomes derived from BMSCs. Briefly, BMSCs were seeded in a 10-cm cell culture dish, and after the cells had grown to the logarithmic phase (about 10^7^ cells/dish), exosome-depleted FBS medium was replaced, and the supernatant was collected 48 h later. The collected culture medium was centrifuged at 300 g for 10 min, and the supernatant was removed to remove cell precipitates. The cells were then centrifuged at 2000 g for 10 min to remove dead cells. This was followed by centrifugation at 10,000 g for 30 min to remove cell debris. The exosomes were then centrifuged at 100,000 × g for 2 h to collect the precipitate. The exosome precipitate was resuspended in 1 × PBS and stored in a −80 °C refrigerator.

Exosomes were counterstained with 3% (w/v) sodium phosphotungstate solution for 5 min at room temperature, and photographed with TEM (Delong America, Montreal, QC, Canada). The diameter and size distribution of exosomes were measured by NTA (NanoSight; Malvern Panalytical, Worcestershire, UK).

### 2.4. Western blot

RIPA lysis buffer (Thermo) was utilized to extract total protein from exosomes and HUVECs. BCA kit (Thermo Scientific, USA) was applied to determine total protein concentration. We used 10% SDS-PAGE to separate the samples, and then transferred samples to PVDF membranes. Subsequently, we utilized 5% milk to block the membranes at 24 °C temperature and treated with anti-CD63 primary antibodies (1:1000; Abcam, ab216130), CD9 (1:1000; Proteintech, 20597-I-AP), LFNG (1:5000; Abcam, ab151699), and β-actin (1:10,000; Proteintech, 60008-I-Ig) were incubated overnight at 4 °C. After washing 3 times with TBST buffer, the goat antirabbit IgG H&L (HRP) (1:1000; Beyotime, A0208) and goat antimouse IgG H&L (HRP) (1:1000; Beyotime, A0216) were incubated for 2 h at room temperature. Next, the high sensitive ECL luminescence kit was used for color development, the chemiluminescence imaging analysis system was exposed, and the pictures were collected. Finally, Image J (v5.2.1) was used for densitometric analysis.

### 2.5. Reverse transcription-quantitative polymerase chain reaction (q-PCR)

For q-PCR validation of tsRNA, the total RNA was extracted by TRIzol Reagent (Takara Biotechnology) from plasma exosomes, BMSCs exosomes and HUVECs, respectively. For mRNA, the total RNA was isolated from HUVECs. PrimeScriptTM RT kit and gDNA Eraser kit (TaKaRa, Tokyo, Japan) were utilized to reverse transcribe RNA into cDNA. SYBR Green PCR kit and ABI QuantStudio6 Q6 (Applied Biosystems, Foster City, CA) were applied to test the expression of tsRNA and mRNA, according to the manufacturer’s instruction. Response conditioning was set according to the following procedure: predenaturation at 95 °C for 10 min, 40 cycles of denaturation at 95 °C for 15 s, and annealing at 60 °C for 1 min. U6 (for tsRNA in exosomes and HUVECs) and GAPDH (for mRNA in HUVECs) were the internal parameters of q-PCR (Cheng et al., 2020; Han et al., 2021; Sun et al., 2021; Qu et al., 2022). Three independent experiments were performed. Quantitative analysis was performed by 2^−ΔΔCt^ method. The specific PCR primers are listed in Table S1.

### 2.6. CCK8 assay

The activity of HUVECs was detected by CCK-8 method. The cells were resuspended in a 96-well plate and added 1× 10^3^ cells per well. The cultures were incubated at 37 °C and maintained at 5% CO_2_. Next, 10 μL of CCK-8 solution (Beyotime, Shanghai, China) was added to each well. After incubating at 37 °C, the OD values were detected by a microplate reader (Infinite M1000, TECAN, Switzerland) at 450nm after 1 h. The experiment was repeated 6 times. Cell viability (%) = (A_treatment_−A_blank_)/(A_control_−A_blank_) × 100%.

### 2.7. Cell transfection

Negative control (NC) RNA or synthesized ts-RNA-15797 mimics was mixed with serum-free Opti-MEM (Gibco, Grand Island, NY, USA), respectively, and incubated for 5 min at 24 °C. Then, lipofectamine 2000 reagent (Invitrogen, Carlsbad, CA, USA) was dissolved in serum-free medium Opti-MEM for 5 min at 24 °C, and the above two reagents were mixed. After incubation for 20 min, the mixed reagents were put in BMSCs and HUVECs, respectively. The plates were incubated in a cell incubator at 37 °C, maintained at 5% CO_2_, and then the new medium was replaced after 6 h. Subsequent tests were performed after continuing the incubation for 48 h.

Referring to plasmid construction in other report (Liu et al., 2020), the coding region of LFNG was subcloned into the pcDNA3.1 empty vector to build the pcDNA-LFNG overexpression plasmid. LFNG overexpression was transfected into HUVECs in the same way as described above.

### 2.8. Angiogenesis assay

For angiogenesis assay, HUVECs were incubated with exosomes from BMSCs for 24 h. One h before slab laying, put the 24-well plate on ice, add 10mg/mL matrigel, and incubated at 37 °C for 1 h to polymerize. After the cells were digested and counted, they were seeded on the matrigel-coated well and placed in a cell incubator at 37 °C for one day and maintained at 5% CO_2_. The formed tubes were photographed under an inverted microscope.

### 2.9. Exosome labelling and internalization

DiI lipophilic dye (Invitrogen) tagged with exosomes from BMSCs. They were incubated at 37 °C, the excess dye was washed with PBS after 30 min, and the tagged exosomes were separated again by ultracentrifugation (described above). HUVECs were cultivated with DiI-tagged exosomes for one day, mmobilized with 4% paraformaldehyde for 10 min at 25 °C, washed with PBS, hatched by DAPI (1:500, Invitrogen) for 5 min at 25 °C. Finally, fluorescence microscope was used to observe and photograph.

### 2.10. Transwell assay

The exosomes-treated HUVECs were suspended in serum-free medium and seeded into the upper chambers. The bottom chamber was added the medium containing 10% FBS. After 24 h, the upper chamber liquid was blotted dry and fixed with 800 μL paraformaldehyde for 30 min. Then, 800 μL crystal violet staining solution was used for 30 min. Finally, three random fields were taken under the microscope to count and the results were counted.

### 2.11. RNA sequencing and bioinformatics analysis

RNA sequencing was performed for HUVECs incubated with NC-Exo and tsRNA-15797 mimics-Exo according to the command of the Multiplex RNA Library Preparation Kit (Illumina, USA). Total RNA was extracted from HUVEC by the Trizol method. For each sample, RNA (200 ng) was ligated with a 3 ‘adaptor’, followed by reverse primer hybridization and 5′ adaptor ligation. The prophase product was synthesized into cDNA, followed by PCR enrichment. HiSeq platform (Illumina, USA) was used to sequence the screened DNA fragments. Then, the original sequence data were filtered and mapped to the human genome. DEmRNAs with false discovery rate (FDR) < 0.05 and | log_2_ fold change | >1 was screened by algorithm DESeq2.0. Cluster analysis of DEmRNAs with MEV software draws heat map. The function of DemRNAs was studied by GO annotation and KEGG pathway analysis. RNAHybrid and Miranda software were applied to predict the target genes of tsRNA-15797, and Cytoscape software 3.6.1 was applied to construct tsRNA-mRNAs interaction network analysis.

### 2.12. Luciferase reporter assay

Referring to other study (Zhong et al., 2022), the LFNG fragment predicted to contain the tsRNA-15797 binding site was constructed into the psiCHECK 2 plasmid to construct the WT-LFNG recombinant reporter gene. Meanwhile, this binding site was mutated to obtain the MUT-LFNG recombinant reporter gene. HUVECs were cotransfected with the WT-LFNG reporter plasmid, MUT-LFNG reporter plasmid, pRL-TK Renilla luciferase plasmid, tsRNA-15797 mimics and mimics NC. After 48 h, luciferase activity was measured using a dual-luciferase reporter assay kit (Promega, Madison, WI, USA). Renilla luciferase activity was considered as a control.

### 2.13. Fluorescence in situ hybridization (FISH)

HUVECs were fixed in 4% paraformaldehyde for 10 min at 24 °C, and then the cells were washed three times for 5 min each with PBS. Subsequently, precooled permeabilization solution was added to the cells, incubated for 5 min, and then the permeabilization solution was discarded and the cells were washed with PBS. Next, the cells were cultured in prehybridization solution (Blocking Solution: Prehybridization Buffer = 1: 99) for 30 min at 37 °C. At the same time, 20 μM tsRNA-15797 (3′-FITC) and LFNG (3′Cy3) probes were added to the hybridization solution (Blocking Solution: Hybridization Buffer = 1: 99) under dark conditions. Subsequently, the prehybridization solution was discarded and the hybridization solution containing the probe was added and incubated in the dark at 37 °C overnight. Cells were washed sequentially with hybrid wash I/II/III and finally with PBS under dark conditions. DAPI was used to counterstain the nuclei. Images were acquired by fluorescence microscopy.

### 2.14. Statistical analysis

Statistical analysis was performed for SPSS 21.0 software (IBM, USA). Data are expressed as mean ± SD. Student’s t test was applied to compare the means between the two groups. Multiple comparisons were tested by one-way analysis of variance. p < 0.05 was regarded as statistically significant.

## 3. Results

### 3.1. The low level of angiogenesis-related tsRNA-15797 in plasma-derived exosomes from ONFH patients

**Figure 1:**
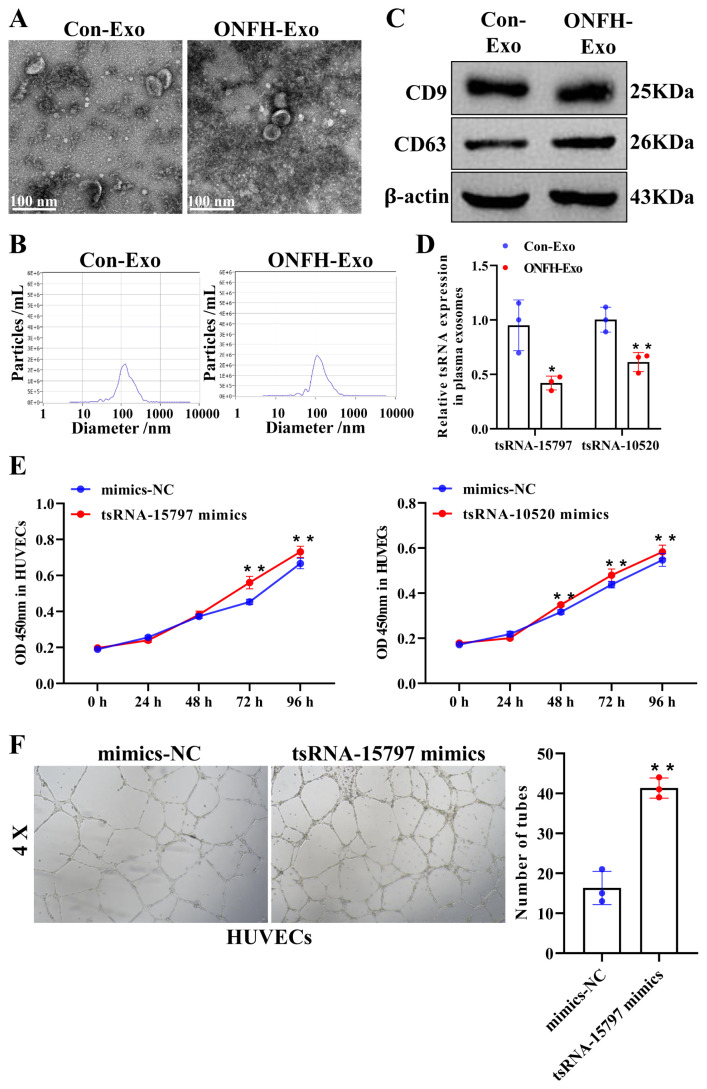
Angiogenesis-related tsRNA-15797 is low expressed in plasma exosomes of ONFH patients. A, Representative electron micrograph of exosomes (scale bars = 100 nm). B, The NTA was used to measure the size, distribution, and content of plasma exosomes. Y-axis: the content of exosomes/ml. C, Western blot analysis showing the presence of CD9 and CD63 in exosomes derived from the BMSCs. D, The expression of tsRNA in exosomes was determined by q-PCR. E, CCK8 was used to detect the effect of tsRNA on cell viability of HUVECs. F, Effect of exosomes derived from tsRNA-15797 mimics on tube formation ability of HUVECs. *p < 0.05, **p < 0.01.

We have previously found that 86 exosomal tsRNA was down-regulated in the plasma of ONFH patients compared with the healthy individuals by small RNA sequencing (Fang et al., 2020). To further investigate the molecular mechanism of exosomal tsRNA involved in angiogenesis of ONFH, we finally screened tsRNA-15797 and tsRNA-10520, according to the evidence that their target genes were involved in angiogenesis-related pathways, including HIF-1α signaling pathway (Zhang et al., 2018), VEGF signaling pathway (Liang et al., 2021), Wnt signaling pathway (Jiang et al., 2015) and TGF-beta signaling pathway (Wang et al., 2013) (Figure S1). Subsequently, to further verify the expression of tsRNA-15797 and tsRNA-10520 in plasma exosomes from ONFH patients, we first isolated exosomes derived from plasma of ONFH patients and healthy subjects, and comprehensively characterized the morphologies and structures of exosomes by TEM and NTA methods. The results of TEM showed that the plasma particles have double membrane structure, which are mainly round or cup shaped (Figure 1A). NTA showed that the mean particles size of ONFH was 121.9 nm and the average concentration was 5.4 × 10^11^ particles/mL, while the particle size of healthy controls was 125.0 nm and the average concentration was 5.2 × 10^11^ particles/mL (Figure 1B). Moreover, western blot showed that CD9 and CD63, exosome markers, were all expressed in ONFH patients- and healthy subjects-derived exosomes, suggesting the successful extraction and purification of exosomes (Figure 1C). Next, we examined their expression in exosomes. Not surprisingly, they were all down-regulated in ONFH-exosomes (Figure 1D). To verify the correlation between tsRNA-15797 and tsRNA-10520 and angiogenesis, we transfected mimics of these two tsRNAs in HUVECs, which exhibited that both tsRNAs promoted endothelial cell viability (Figure 1E). Since tsRNA-15797 was the most down-regulated and had the greatest effect on endothelial viability, we chose tsRNA-15797 for subsequent study. Angiogenesis assay showed that compared with the mimics-NC, tsRNA-15797 obviously promoted vascular angiogenesis (Figure 1F). These results suggested that tsRNA-15797 is down-expressed in exosomes derived from plasma of ONFH patients and promotes angiogenesis of HUVECs.

### 3.2. tsRNA-15797-modified BMSC-derived exosomes promote angiogenesis of HUVECs

**Figure 2:**
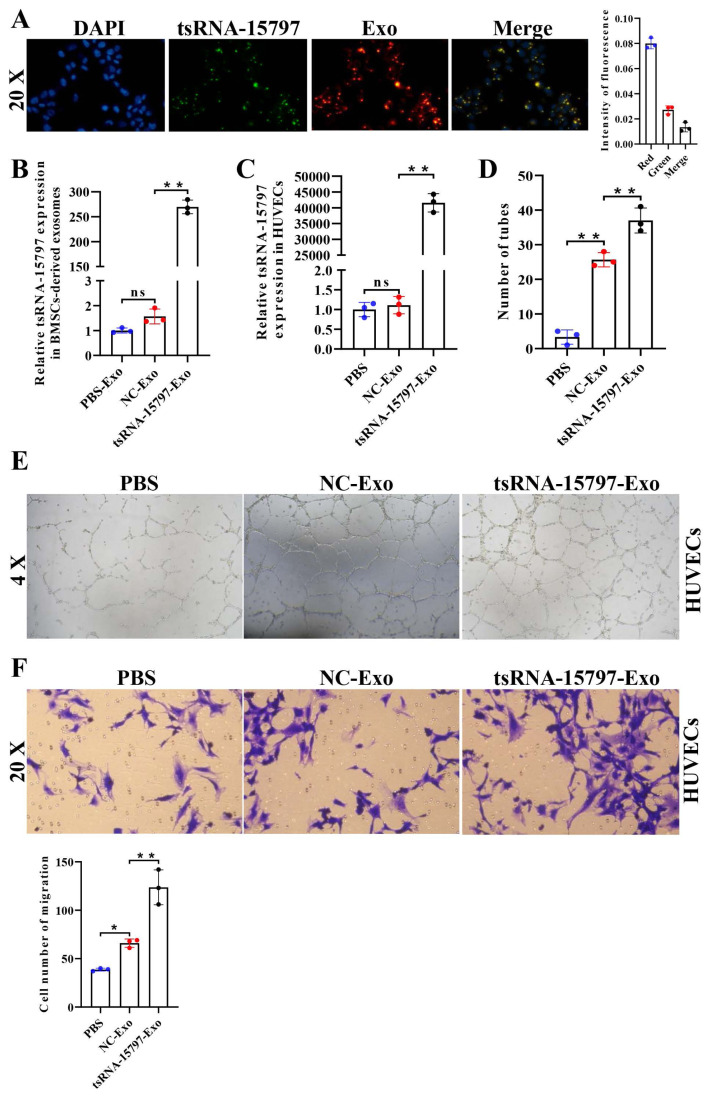
tsRNA-15797-modified BMSCs exosomes promote angiogenesis of vascular endothelial cells. A, DiI-exosomes(red)and 5(6)-FAM-tsRNA-15797(green) were observed by immunofluorescence. B, The expression of tsRNA-15797 in exosomes derived from BMSCs treated with PBS-NC or tsRNA-15797 mimics was determined by q-PCR. C, The expression of tsRNA-15797 in vascular endothelial cells cocultured with tsRNA-15797 mimics-exo was assessed by qPCR. D, The number of tubes was detected using tube formation. E, Representative pictures of the tube formation experiment. F, Endothelial cell migration was analyzed by transwell assay. **p < 0.01, ns means no significance.

BMSC-derived exosomes have therapeutic effects in various diseases such as osteonecrosis, which has been demonstrated in our previous study (Fang et al., 2020). Recently, some studies have shown that RNA-modified BMSCs-derived exosomes can be used as therapeutic tools for various diseases (Yang et al., 2019). Given the low expression of tsRNA-15797 in plasma exosomes from ONFH patients and its promotion in angiogenesis, we further evaluated the potential of tsRNA-15797-modified BMSCs-derived exosomes as therapeutic tools for ONFH by studying their regulatory effects on angiogenesis. We first transfected human BMSCs with Cy3-labeled tsRNA-15797, and extracted exosomes for Dil labeling. After incubation of endothelial cells with exosomes, we observed that BMSCs-derived exosomes carried tsRNA-15797 into HUVECs (Figure 2A). Next, tsRNA-15797 mimics were transfected into BMSCs and the exosomes were isolated, demonstrating successful construction of tsRNA-15797-modified exosomes by q-PCR method verification (Figure 2B). We incubated HUVECs with the different exosomes, and divided HUVECs into three groups: PBS group, NC-Exo group, and tsRNA-15797 mimics-Exo group. The results of q-PCR showed there was no difference in the expression of tsRNA-15797 between PBS and NC-Exo while the expression of tsRNA-15797 in tsRNA-15797-Exo group was higher than that in NC-Exo (Figure 2C). Angiogenesis and transwell assay were then performed to explore whether exosomal tsRNA-15797 regulates endothelial cell angiogenesis and migration. Compared with the PBS group, NC-Exo group obviously enhanced vascular angiogenesis and ability to traverse HUVECs, whereas tsRNA-15797 overexpression had a stronger result (Figure 2D–F). These data advised that tsRNA-15797-modified BMSC exosomes could effectively induced angiogenesis.

### 3.3. Global landscape of target genes regulated by tsRNA-15797

**Figure 3:**
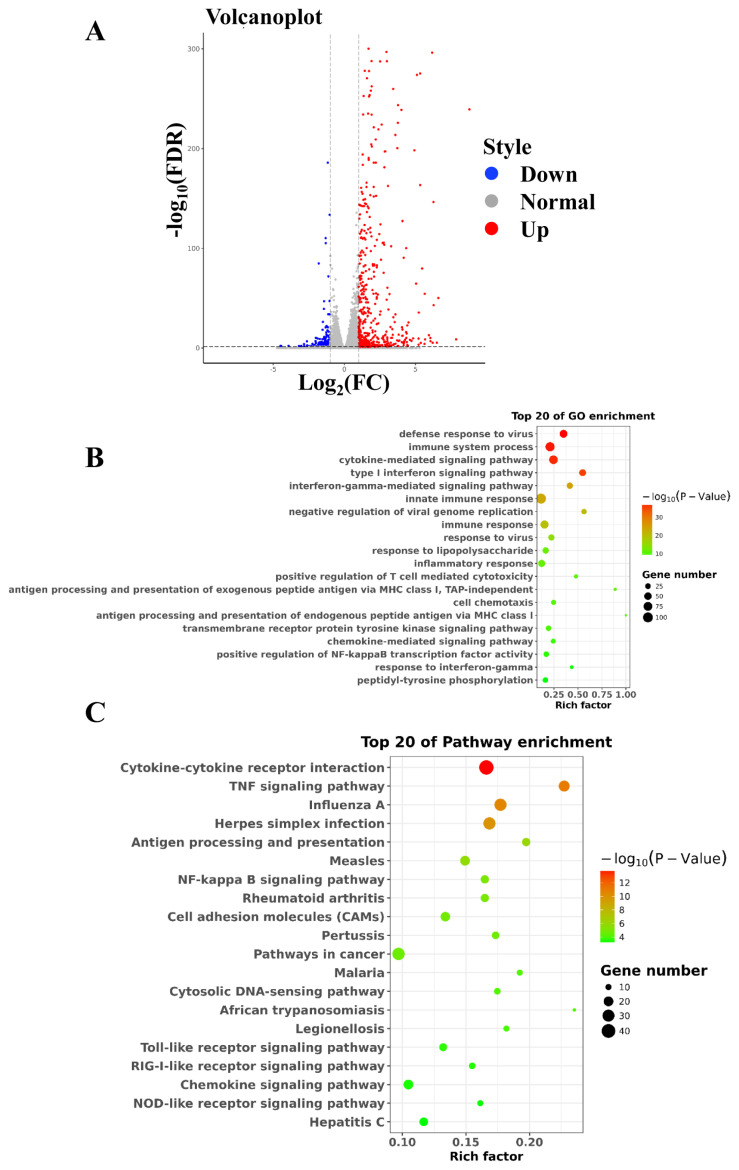
Functional and pathway analysis of tsRNA-15797. A, Volcano plot showing the differentially expressed targeted genes. B, The top 20 enriched GO terms of the differentially expressed targeted genes. C, The top 20 enriched KEGG pathways of the differentially expressed targeted genes. Rich factor included the gene numbers and P-values.

To identify the molecular mechanism of the induction of angiogenesis mediated by tsRNA-15797-modified exosomes, RNA sequencing was performed on HUVECs incubated with NC-Exo and tsRNA-15797-Exo. A total of 844 differently expressed mRNAs (| log_2_ fold change | >1 and FDR value < 0.05) were identified in HUVECs between NC-Exo and tsRNA-15797-Exo (Figure 3A). Functional enrichment analysis identified that these differentially expressed mRNAs were significantly enriched in biological functions related to immune, cell migration and adhesion, such as “immune system process”, “cytokine-mediated signaling pathway”, “cell chemotaxis”, and “chemokine-mediated signaling pathway” (Figure 3B). The KEGG pathway indicated these differentially expressed mRNAs involved in the signaling pathway of “Cell adhesion molecules (CAMs)”, “NF-κB signaling pathway”, “Toll like signaling pathway”, “NOD-like receptor signaling pathway”, and “chemokine signaling pathway” (Figure 3C).

### 3.4. tsRNA-15797 inhibits LFNG expression

**Figure 4:**
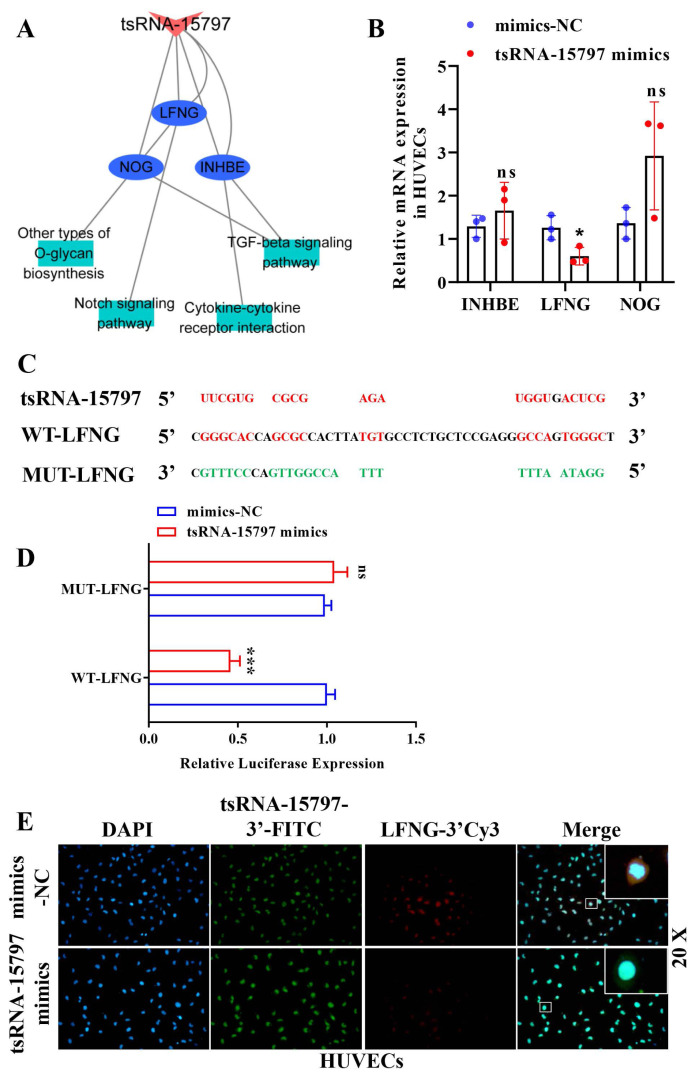
tsRNA-15797 interacts with LFNG. A, Network diagram showing the tsRNA-15797-mRNA-pathway. B, The expression of mRNA in HUVECs incubated with tsRNA-15797-modified BMSCs exosomes was determined by q-PCR. C, Putative tsRNA-15797 binding site on LFNG mRNA. Red represents the putative site and green represents the mutated site. D, The relative luciferase activity of the WT-LFNG reporter and MUT-LFNG in HUVECs transfected with tsRNA-15797 mimics. E, FISH assay was used to detect the colocalization of tsRNA-15797 and LFNG in HUVECs. *p < 0.05, **p < 0.01, ns means no significance.

In order to investigate how tsRNA-15797 exerted its function in ONFH progression, INHBE, LFNG, and NOG were chosen as candidate target genes of tsRNA-15797, according to down-regulated mRNAs and the predicted target genes of tsRNA-15797. According to the biological functions of different genes, we constructed the network interaction diagram of tsRNA-15797-mRNA-pathway (Figure 4A). These mRNAs are primarily involved in “Notch signaling pathway”, “TGF-beta signaling pathway”, and “cytokine-cytokine receptor interaction”. To validate our sequencing data, we performed q-PCR validation. The q-PCR indicated that only LFNG was significantly down-regulated in HUVECs after tsRNA-15797 overexpression (Figure 4B). Luciferase reporter assay confirmed the direct interaction between tsRNA-15797 and LFNG (Figure 4C and D). Similarly, FISH analysis showed that both tsRNA-15797 and target genes localized to the cytoplasm (Figure 4E). The result suggested that tsRNA-15797 negatively regulated LFNG expression in HUVECs.

### 3.5. tsRNA-15797-modified BMSC exosomes promote angiogenesis of HUVECs by targeting down-regulated LFNG

**Figure 5:**
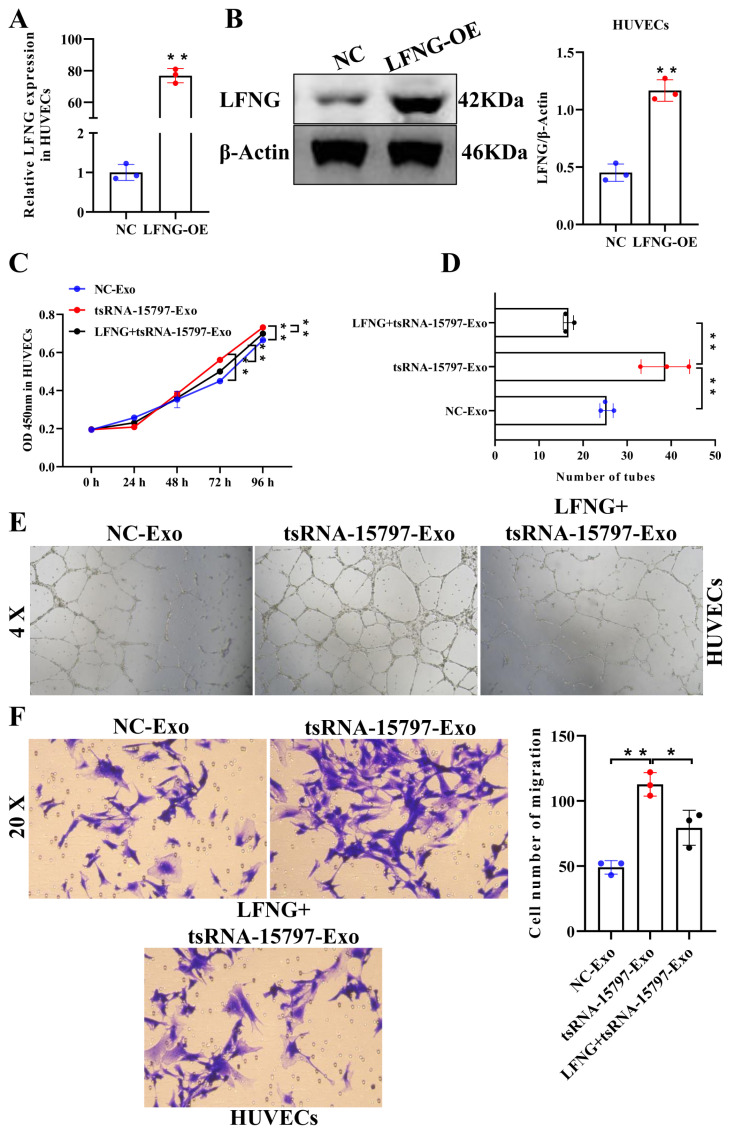
tsRNA-15797-modified BMSC exosomes promote angiogenesis of HUVECs through LFNG. Transfection efficiency of LFNG overexpression into HUVECs was determined by q-PCR (A) and western blot (B). C, CCK8 was used to detect the cell viability of HUVECs. D, The number of tubes was detected using tube formation. E, Representative pictures of the tube formation experiment. F, Endothelial cell migration was analyzed by transwell assay. *p < 0.05, **p < 0.01, ns means no significance.

To further examine the molecular mechanism of tsRNA-15797-modifed exosomes regulating angiogenesis of HUVECs, we first constructed LFNG overexpression vector (Figure 5A and B). Then, we cocultured HUVECs with tsRNA-15797-modifed exosomes and LFNG overexpression vector. The endothelial cell viability was increased after expression of tsRNA-15797, and reduced after further transfection with LFNG overexpression vector (Figure 5C). Similarly, LFNG reversed the effect of tsRNA-15797-modifed exosomes on the angiogenesis of HUVECs (Figure 5D and E). Furthermore, the cell migration was promoted by tsRNA-15797-modifed exosomes and further reduced by overexpression of LFNG (Figure 5F). In summary, we suggested that LFNG could potentially play a role in angiogenesis of HUVECs that induced by tsRNA-15797-modified BMSCs-derived exosome.

## 4. Discussion

ONFH is a progressive debilitating disease with femoral head structural changes, collapse, deformation, joint inflammation, and dysfunction (George and Lane, 2022). Our previous study found that tsRNA-modified BMSC-derived exosomes boosted the osteogenic differentiation ability (Fang et al., 2020). However, the exact mechanism involved remains unclear. Therefore, we revealed the molecule molecular mechanism of tsRNA-modified BMSC-derived exosomes in angiogenesis of ONFH. We demonstrated tsRNA-15797 was observably diminished in the plasma exosomes from ONFH patients and tsRNA-15797-modified BMSC exosomes enhanced angiogenesis and HUVECs migration by targeting down-regulated LFNG.

Emerging studies are beginning to reveal multifunctional roles of tsRNA in biological processes, including reverse transformation, gene silencing, and epigenetic inheritance, all based on the RNA modification and protein-binding capacity of tsRNA (Chen et al., 2021). Because of its unique biological role, tsRNA is able to regulate numerous physiological processes. For example, tsRNA-16902 regulated adipogenic differentiation on human mesenchymal stem cells by impairing PRARγ via Smad2/3 signaling pathway (Wang et al., 2020). Moreover, abnormal expression of tsRNA is associated with various human disease processes and can be used as a biomarker for disease diagnosis or treatment (Hu et al., 2021). In this study, we found that tsRNA-15797 was under-expressed in plasma exosomes of ONFH patients and promoted angiogenesis, which indicated tsRNA-15797 has potential as a therapeutic target for ONFH.

Exosomes, as nanoscale vesicles, can be used as drugs for targeted treatment of diseases by themselves and by carrying target molecules (Kalluri and LeBleu, 2020). In contrast to other drug carriers, such as viral vectors and lipid nanoparticles, exosomes can deliver payloads precisely without activating the innate or acquired immune system, making repeated drug delivery much easier (Li et al., 2021). Especially, as a novel therapeutic approach, stem cells-derived exosomes have the promising to be used in tissue damage repair and regeneration as well as in the treatment of other diseases by promoting cell proliferation and differentiation, and inhibiting cell inflammation (Ha et al., 2020; Han et al., 2021). In addition, stem cells-derived exosomes have been confirmed to be involved in angiogenesis, such as mesenchymal stem cells-derived exosomes facilitated angiogenesis to promote cutaneous wound healing (Zhang et al., 2015). Lu et al. reported that exosomes derived from BMSCs can be absorbed by HUVECs and facilitated the migration and tube formation of HUVECs (Lu et al., 2020). Furthermore, BMSCs-secreted exosomal small RNA into brain microvascular endothelial cells, thereby promoting angiogenesis and leading to ameliorate the ischemic brain injury (Hou et al., 2020). Consistent with these studies, we certified tsRNA-15797-overexpressed BMSCs-derived exosomes ameliorate ONFH by boosting the angiogenesis of HUVECs.

In order to understand how tsRNA-15797-modified exosomes regulate the molecular mechanism of angiogenesis, we performed RNA sequencing of endothelial cells cocultured with tsRNA-15797-overexpressed BMSCs-derived exosomes. Our findings showed there were 844 differently expressed mRNAs in HUVECs between NC-Exo and tsRNA-15797 mimics-Exo. Functional enrichment analysis showed that they focused on “Cell adhesion molecules (CAMs)”, “NF-κB signaling pathway”, and “Toll like signaling pathway”. It has been documented that CAMs are involved in some physiological and pathological processes, such as angiogenesis (Francavilla et al., 2009). Moreover, experimental studies in rheumatoid arthritis rats showed that inactivation of Toll-like receptor signaling pathway inhibited inflammatory response and angiogenesis (Hu et al., 2019). These results suggested that exosomes modified by tsRNA-15797 affect angiogenesis by affecting expression of mRNA in endothelial cells. Subsequent functional verification experiments proved that LNFG was a target gene negatively regulated by tsRNA-15797, which overexpression of LNFG attenuated the promoting effect of exosomes modified by tsRNA-15797 on endothelial cell angiogenesis.

## 5. Conclusion

Collectively, our results first provided the molecular mechanism of tsRNA-15797-modified exosomes regulated angiogenesis of ONFH. We demonstrated that tsRNA-15797-modified BMSC-derived exosomes improved the angiogenesis potency of HUVECs by targeting to inhibit LNFG. This study expands the application of tsRNA and BMSC-derived exosomes in ONFH treatment.

## Supplementary material

Figure S1 Sankey Diagram showing the relationship of tsRNA-mRNA-angiogenesis-related pathways.

**Table S1:** Sequence information for specific primers.

Primer	Primer sequence (5′to3
tsRNA-10520-RT	GTCGTATCCAGTGCGTGTCGTGGAGTCGGCAATTGCACTGGATACGACAAGCGAG
tsRNA-15797-RT	GTCGTATCCAGTGCGTGTCGTGGAGTCGGCAATTGCACTGGATACGACAAGCAC
U6-F	CGATACAGAGAAGATTAGCATGGC
U6-R	AACGCTTCACGAATTTGCGT
INHBE-F	CTCTGCCCTCTAGTGGCTTGA
INHBE-R	CGCCTCGGTTGTCCAGTAA
LFNG-F	GTCAGCGAGAACAAGGTGC
LFNG-R	GATCCGCTCAGCCGTATTCAT
NOG-F	CATCGAACACCCAGACCCTA
NOG-R	CATGAAGCCTGGGTCGTAGT
GAPDH-F	ACAACTTTGGTATCGTGGAAGG
GAPDH-R	GCCATCACGCCACAGTTTC

## Data Availability

The datasets used and/or analyzed during the current study are available from the corresponding author on reasonable request.
