# Supplementary figures and images for: tsRNA-15797-modified BMSC-derived exosomes mediate LFNG to induce angiogenesis in osteonecrosis of the femoral head
Source: Turk J Biol. 2023 May 18;47(3):186–98. doi: 10.55730/1300-0152.2654 (PMC10388130; doi:10.55730/1300-0152.2654)

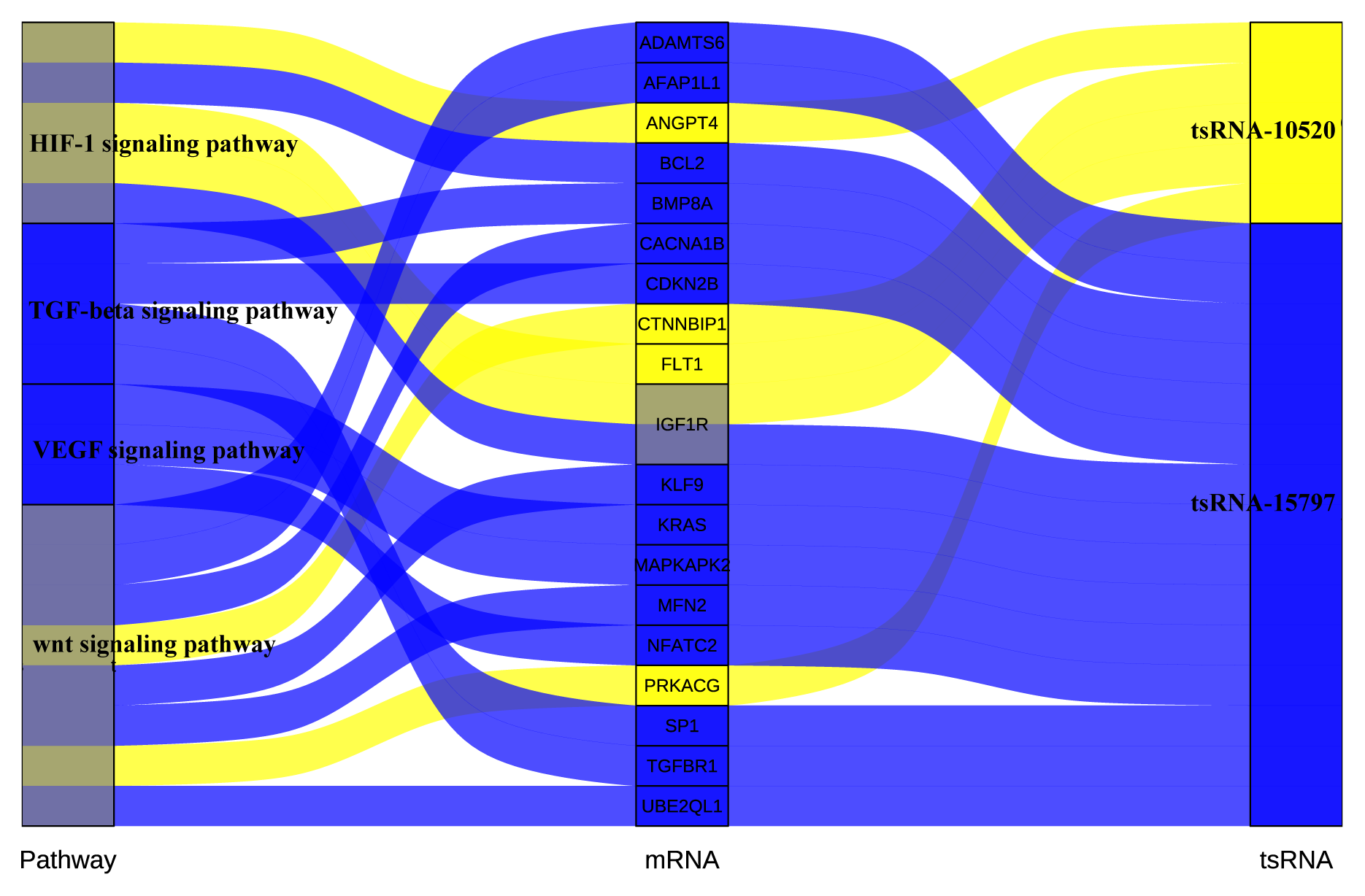

Supplement: Figure S1 — Sankey Diagram showing the relationship of tsRNA-mRNA-angiogenesis-related pathways. [file turkjbiol-47-3-186s1.tif]
